# Evaluation of Changes in Tumor Shadows and Microcalcifications on Mammography Following KORTUC II, a New Radiosensitization Treatment without any Surgical Procedure for Elderly Patients with Stage I and II Breast Cancer

**DOI:** 10.3390/cancers3033496

**Published:** 2011-09-09

**Authors:** Akira Tsuzuki, Yasuhiro Ogawa, Kei Kubota, Shiho Tokuhiro, Ryo Akima, Shin Yaogawa, Kenji Itoh, Yoko Yamada, Toshikazu Sasaki, Masahide Onogawa, Tomoaki Yamanishi, Shinji Kariya, Munenobu Nogami, Akihito Nishioka, Mitsuhiko Miyamura

**Affiliations:** 1 Department of Diagnostic Radiology & Radiation Oncology, Medical school, Kochi University Nankoku, Kochi 783-8505, Japan; E-Mails: jm-tsuzukia@kochi-u.ac.jp (A.T.); kubotak@kochi-u.ac.jp (K.K.); jm-tokuhiros@kochi-u.ac.jp (S.T.); jm-akima.r@kochi-u.ac.jp (R.A.); jm-yaogawa@kochi-u.ac.jp (S.Y.); jm-itohk@kochi-u.ac.jp (K.I.); jm-yohko-y@kochi-u.ac.jp (Y.Y.); jm-sasakis@kochi-u.ac.jp (T.S.); yamanist@kochi-u.ac.jp (T.Y.); kariyas@kochi-u.ac.jp (S.K.); mnogami@kochi-u.ac.jp (M.N); nishiokaa@kochi-u.ac.jp (A.N.); 2 Department of Pharmacy, Medical School, Kochi University, Nankoku, Kochi 783-8505, Japan; E-Mails: jm-ma_ono@kochi-u.ac.jp (M.O.); miyamus@kochi-u.ac.jp (M.M.)

**Keywords:** mammography, breast cancer, KORTUC, hydrogen peroxide, radiosensitizer, sodium hyaluronate

## Abstract

We introduced non-surgical therapy with a novel enzyme-targeting radiosensitization treatment, Kochi Oxydol-Radiation Therapy for Unresectable Carcinomas, Type II (KORTUC II) into early stages breast cancer treatment. The purpose of this study was to examine changes in tumor shadows and microcalcifications on mammography (MMG) following KORTUC II for elderly patients with breast cancer. We also sought to determine whether MMG was useful in evaluating the therapeutic effect of KORTUC II. In addition to MMG, positron emission tomography-computed tomography (PET-CT) was performed to detect both metastasis and local recurrence. In all 10 patients, tumor shadows on MMG completely disappeared in several months following the KORTUC II treatment. The concomitant microcalcifications also disappeared or markedly decreased in number. Disappearance of the tumors was also confirmed by the profile curve of tumor density on MMG following KORTUC II treatment; density fell and eventually approached that of the peripheral mammary tissue. These 10 patients have so far have also shown neither local recurrence nor distant metastasis on PET-CT with a mean follow-up period of approximately 27 months at the end of September, 2010. We conclude that breast-conservation treatment using KORTUC II, followed by aromatase inhibitor, is a promising therapeutic method for elderly patients with breast cancer, in terms of avoiding any surgical procedure. Moreover, MMG is considered to be useful for evaluating the efficacy of KORTUC II.

## 1. Introduction

In recent years, local therapeutic procedures for breast cancer have been expected to be minimally invasive on the basis that permanent curability is estimated to be comparable to that of wider resections. Therefore, we introduced non-surgical therapy with a novel enzyme-targeting radiosensitization treatment, Kochi Oxydol-Radiation Therapy for Unresectable Carcinomas, Type II (KORTUC II) [1] into early stages breast cancer treatment. Using KORTUC II, low linear energy transfer (LET)-radioresistant tumors can be converted into radiosensitive ones; this has been already demonstrated based on both our experimental results which showed hydrogen peroxide to be a strong radiosensitizer [2-5] and our clinical studies [1,6-9].

The purpose of this study was to examine changes in tumor shadows and microcalcifications on mammography (MMG) following KORTUC II for elderly patients with breast cancer. We also sought to determine whether MMG was useful in evaluating the therapeutic effect of KORTUC II. In addition to MMG, positron emission tomography-computed tomography (PET-CT) was performed to detect both metastasis and local recurrence.

## 2. Results

Patient data are summarized in Table 1. In all 10 patients, tumor shadows on MMG completely disappeared in several months following the KORTUC II treatment. The concomitant microcalcifications also disappeared or markedly decreased in number. These data are shown in Tables 2 and 3.

**Figure 1.**
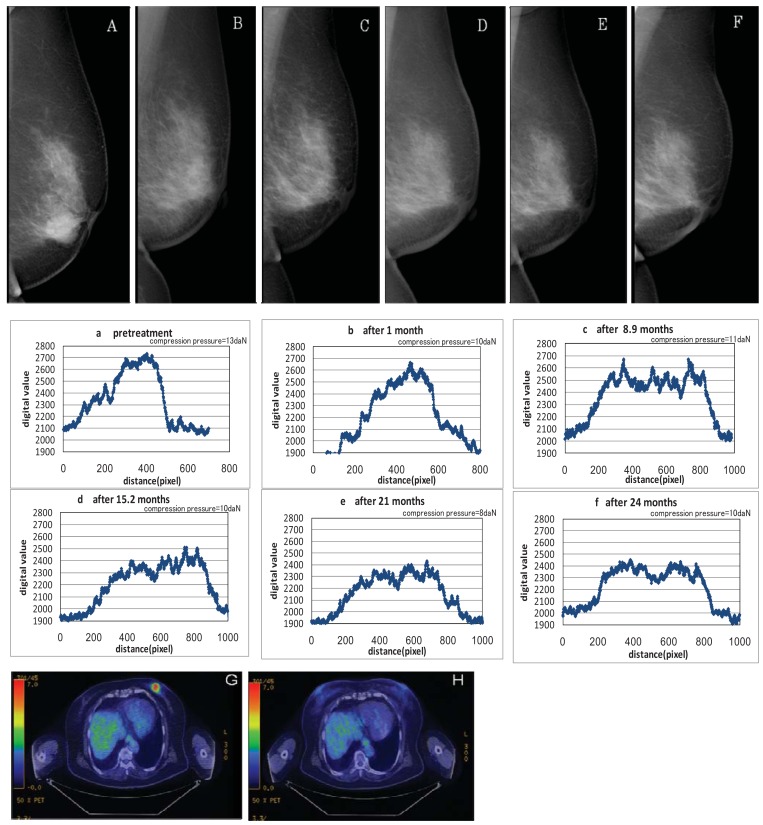
Case 2. Aged patient (79F) with left breast cancer (cT2N0M0) treated with radiosensitization (KORTUC II). A ∼ F: Pre and post-KORTUC II treatment evaluation of changes of tumor shadows on MMG. a ∼ f: Profile curve of changes of tumor shadows (1 pixel = 93.9 μm). G, H: PET-CT images before KORTUC II (G) and 13 months after KORTUC II (H). (G) Tumor diameter is 22 mm (SUV_MAX_ = 7.4). (H) There is neither local recurrence nor distant metastasis.

**Figure 2.**
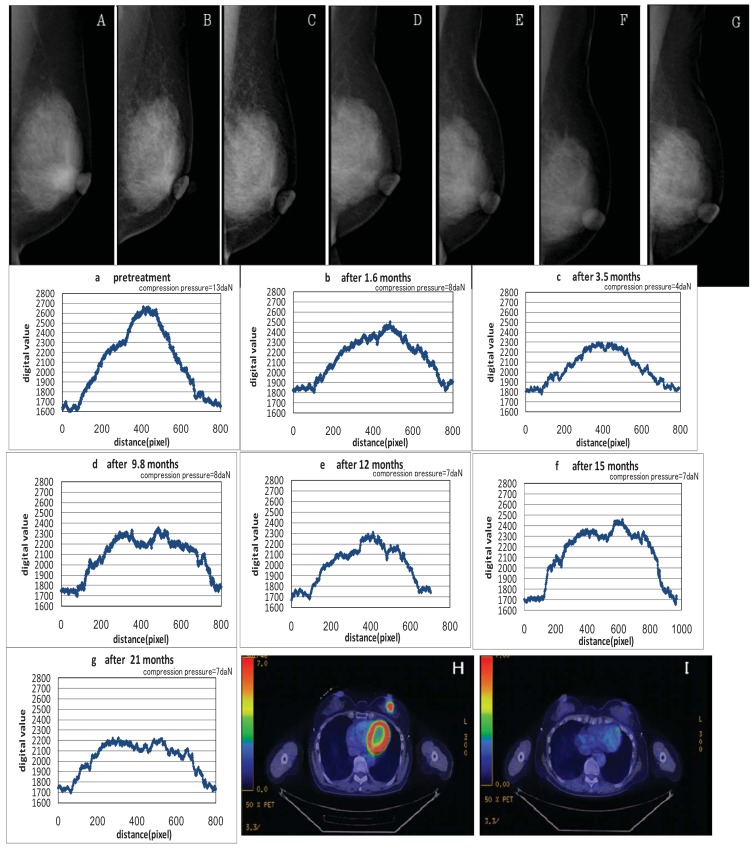
Case 4. 59 years old female patient with left breast cancer (cT2N0M0) treated with radiosensitization (KORTUC II). A ∼ G: Pre and post-KORTUC II treatment evaluation of changes of tumor shadows on MMG. a ∼ g: Profile curve of changes of tumor shadows (1 pixel = 93.9 μm). H, I: PET-CT images before KORTUC II (H) and 28 months after KORTUC II (I). (H) Tumor diameter is 27 mm (SUV_MAX_ = 8.1). (I) There is neither local recurrence nor distant metastasis.

**Figure 3.**
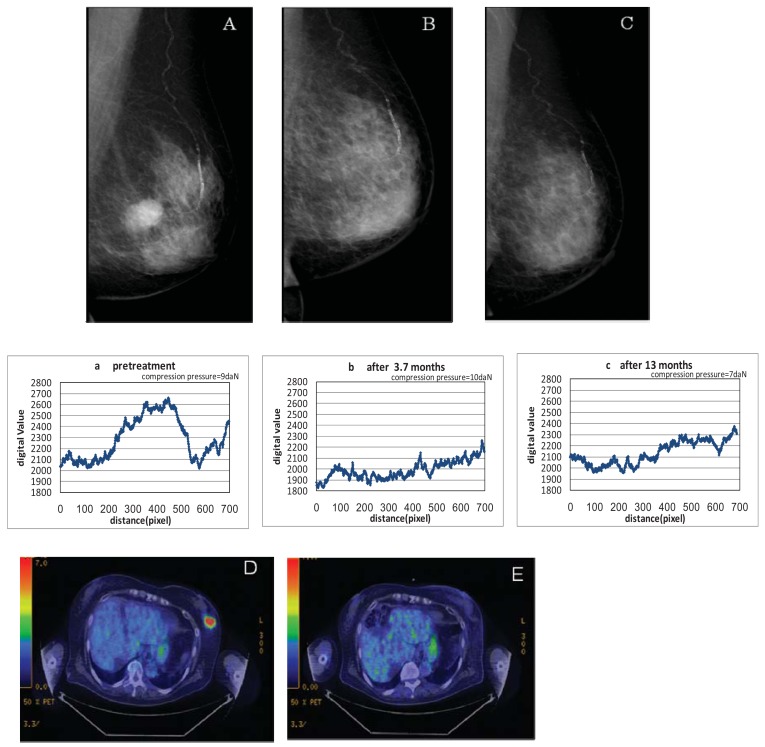
Case 6. Aged patient (79F) with left breast cancer (cT2N0M0) treated with radiosensitization (KORTUC II). A ∼ C: Pre and post-KORTUC II treatment evaluation of changes of tumor shadows on MMG. a ∼ c: Profile curve of changes of tumor shadows (1 pixel = 93.9 μm). D, E: PET-CT images before KORTUC II (D) and 4.6 months after KORTUC II (E). (D) Tumor diameter is 30 mm (SUV_MAX_ = 8.4). (E) There is neither local recurrence nor distant metastasis.

Disappearance of the tumors was also confirmed by the profile curve of tumor density on MMG following the KORTUC II treatment; density fell and eventually approached that of the peripheral mammary tissue. The examples of changes in size of tumor shadows and microcalcifications are shown in Figures 1, 2, and 3.

These 10 patients have so far shown neither local recurrence nor distant metastasis also on PET-CT with a mean follow-up period of approximately 27 months at the end of September, 2010.

## 3. Discussion

Recently, breast-conserving surgery has become the most common surgical procedure for breast cancer treatment. However, even this type of surgery often has an unacceptable cosmetic outcome. Therefore, various types of minimally invasive options have been used as alternatives to surgical therapy, such as radiofrequency ablation (RFA), focused ultrasound ablation (FUS), and cryotherapy. These minimally invasive approaches are currently being investigated. Although excellent locoregional control can be obtained with these procedures, long-term control rates remain unknown. Moreover, RFA and cryotherapy demand insertion of a relatively large needle into the breast. General anesthesia is essential to perform RFA, and FUS is very time-consuming and requires MRI to monitor the thermal distribution. It is also important to note that these non-surgical approaches require adjuvant radiation therapy to non-ablated tissue in order to eliminate residual cancerous tissue.

KORTUC II, a form of radiation therapy intensified with a newly-developed radiosensitizer for intratumoral injection, is a logical technique for the ablation of cancerous nests throughout the breast. KORTUC II has an advantage over other non-surgical ablation therapies, as it can treat breast cancer with one treatment. General anesthesia, insertion of a large needle, and expensive equipment to monitor thermal distribution are unnecessary with this method.

Precise assessment of therapeutic efficacy is important when evaluating breast cancer treatments. MMG has been reported to be reliable for the detection of breast tumors and microcalcifications. Therefore, this study used MMG and PET-CT as diagnostic tools for the assessment of the therapeutic effects of KORTUC II on primary breast tumors. Tumors disappeared on MMG in several months following KORTUC II treatment. Moreover, PET-CT confirmed that the absence of recurrence and distant metastasis. Therefore, KORTUC II is considered to be an ideal non-surgical treatment for elderly patients with early stages breast cancer.

## 4. Patients and Methods

Ten elderly female patients with breast cancer (invasive ductal carcinoma) but no clinical evidence of distant metastasis were enrolled in the KORTUC II trial. Each patient signed an informed consent form before participation in the study. Patient data are summarized in Table 1. Patients were eligible for this study if they had contraindications to general anesthesia due to old age and/or significant cormorbidity, or they had declined surgical treatment.

For each patient, radiation therapy (RT) with 4 MV X-ray was delivered with an EXL-20TP linear accelerator equipped with a multileaf collimator (Mitsubishi Electric Co. Ltd., Tokyo, Japan) [10,11]. Hypofractionated RT was administered using a tangential field approach & a Field-in-field method: the total dose was 44 Gy administered as 2.75 Gy/fraction. RT was performed five times per week for each patient. Boost irradiation was delivered using an electron beam of appropriate energy for each individual patient and was administered concurrently with a dose of 9 Gy in three fractions in the last week of RT with 4 MV X-ray.

The new radiosensitizer was injected into the breast tumor tissue twice a week under ultrasonographic guidance, just prior to each administration of RT from the 6^th^ fraction onwards. At the injection of the agent, small amount of 1% lidocaine hydrochloride was used for pain relief at the injection site. The agent is composed of 0.5% hydrogen peroxide and 0.83% sodium hyaluronate, which is safe for injection and effectively preserves oxygen concentration in the tumor tissue for more than 24 hours following intratumoral injection [12]. For sodium hyaluronate, we used one syringe (2.5 mL) of a hyaluronic acid preparation having a 1% w/v concentration of sodium hyaluronate (ARZ Dispo, Seikagaku Corporation, Tokyo, Japan). To this, 0.5 mL of a 3% w/v solution of hydrogen peroxide in a vial subdivided aseptically at the pharmacy department of our hospital (Oxydol, Ken-ei Pharmaceutical Co. Ltd., Osaka, Japan) was added immediately before use, and mixed well to prepare the radiosensitizer.

Hormonal status was examined on a specimen obtained by needle biopsy at pre-treatment. Patients with estrogen receptor-positive tumors also underwent hormonal therapy using aromatase inhibitor for five years following KORTUC II. MMG was performed approximately twice a year during follow-up according to the Japanese Standard Methods established by the Japanese Central Committee on Quality Control of Mammographic Screening.

The therapeutic effects of KORTUC II were evaluated in terms of the changes in size of tumor shadows and microcalcifications shown on the monitor (3M pixel, Nanao Co. Ltd., Japan) before and following KORTUC II. Changes of tumor density on MMG were also analyzed using image analysis software image-J (National Institutes of Health, Bethesda, Maryland, USA). PET-CT was also performed annually during follow-up to detect both distant metastasis and local recurrence.

## 5. Conclusions

We conclude that breast-conservation treatment using KORTUC II followed by aromatase inhibitor is a promising therapeutic method for elderly patients with breast cancer, in terms of avoiding any surgical procedure. Moreover, MMG is considered to be useful for evaluating the efficacy of KORTUC II.

## Figures and Tables

**Table 1.** Therapeutic effect of the radiosensitization treatment (KORTUC II) for breast cancer.

**Case**	**Diseased site**	**TNM class**	**Age/gender**	**Therapeutic effect**	**Side effects**

1	right	cT2N0M0	88F	CR, NED>39 months	mild skin burn
2	left	cT2N0M0	79F	CR, NED>38 months	mild skin burn
3	right	cT1cN0M0	79F	CR, NED>38 months	mild skin burn
4	left	cT2N0M0	59F	CR, NED>38 months	mild skin burn
5	left	cT1cN0M0	73F	CR, NED>30 months	mild skin burn
6	left	cT2N0M0	79F	CR, NED>20 months	mild skin burn
7	right	cT1cN0M0	77F	CR, NED>20 months	mild skin burn
8	right	cT2N0M0	82F	CR, NED>19 months	mild skin burn
9	left	cT2N0M0	63F	CR, NED>15 months	mild skin burn
10	right	cT1cN0M0	77F	CR, NED>38 months	mild skin burn

**Table 2.** Changes of tumor shadows following the radiosensitization treatment (KORTUC II).

**Case**	**Time**			
1	**Pretreatment**	**After 3.8 mo.**	**After 8 mo.**	**After 27.6 mo.**
	Shape is round Border is microlobulated High density tumor	Disappearance	Disappearance	Disappearance
2	**Pretreatment**	**After 1 mo.**	**After 8.9 mo.**	**After 15.2∼30 mo.**
	Shape is oval Border is microlobulated High density tumor	Disappearance	Disappearance	Disappearance
3	**Pretreatment**	**After 1 mo.**	**After 8.9 mo.**	**After 15.2∼30 mo.**
	Shape is round Border is microlobulated High density tumor	Disappearance	Disappearance	Focal asymmetric density (FAD) Skin retraction
4	**Pretreatment**	**After 1.6 mo.**	**After 3.5 mo.**	**After 9.8∼21 mo.**
	Shape is round Border is microlobulated High density tumor	Reduction	Shape is indistinct Architectural distorsion	Disappearance
5	**Pretreatment**	**After 8.6 mo.**	**After 12.6 mo.**	
	Shape is oval Border is spiculated High density tumor	Disappearance	Disappearance	
6	**Pretreatment**	**After 3.7 mo.**	**After 13 mo.**	
	Shape is oval Border is microlobulated High density tumor	Disappearance	Disappearance	
7	**Pretreatment**	**After 5.9 mo.**	**After 13.6 mo.**	**After 19.4 mo.**
	Pleomorphic tumor Shape is irregular Border is microlobulated High density tumor	Architectural distorsion	Disappearance	Disappearance
8	**Pretreatment**	**After 3 mo.**	**After 12 mo.**	
	Shape is oval Border is spiculated High density tumor	Spiculated core disappeared Size is unchanged	Disappearance	
9	**Pretreatment**	**After 4.3 mo.**	**After 15.3 mo.**	
	Shape is oval Border is spiculated High density tumor	Shape is irregular Border is microlobulated Equal density (fat-containing)	Disappearance	
10	**Pretreatment**	**After 3 mo.**	**After 14 mo.**	**After 18.6∼33.4 mo.**
	Focal asymmetric density (FAD)	Unchanged from last time	Disappearance	Disappearance

**Table 3.** Changes of microcalcification following the radiosensitization treatment (KORTUC II).

**Case**	**Time**			
1	**Pretreatment**	**After 3.8 mo.**	**After 8 mo.**	**After 27.6 mo.**
	Shape is slightly indistinct Distribution is grouped	More slightly indistinct than last time	Disappearance	Disappearance
2	**Pretreatment**	**After 1 mo.**	**After 8.9 mo.**	**After 15.2∼30 mo.**
	Shape is branching & casting calcification Distribution is grouped	Unchanged	Disappearance	Disappearance
3	**Pretreatment**	**After 1 mo.**	**After 8.9 mo.**	**After 15.2∼30 mo.**
	No calcification	No calcification	No calcification	No calcification
4	**Pretreatment**	**After 1.6 mo.**	**After 3.5 mo.**	**After 9.8∼21 mo.**
	Shape is slightly indistinct Distribution is grouped	Reduction	More reduction	Disappearance
5	**Pretreatment**	**After 8.6 mo.**	**After 12.6 mo.**	
	Shape is small round containing linear Distribution is grouped	Disappearance	Disappearance	
6	**Pretreatment**	**After 3.7 mo.**	**After 13 mo.**	
	Shape is slightly indistinct Distribution is grouped	Disappearance	Disappearance	
7	**Pretreatment**	**After 5.9 mo.**	**After 13.6 mo.**	**After 19.4 mo.**
	Shape is pleomorphic Distribution is segmental	Unchanged	Reduction	Unchanged from last time
8	**Pretreatment**	**After 3 mo.**	**After 12 mo.**	
	Shape is pleomorphic Distribution is grouped	Reduction	More reduction	
9	**Pretreatment**	**After 4.3 mo.**	**After 15.3 mo.**	
	No calcification	No calcification	No calcification	
10	**Pretreatment**	**After 3 mo.**	**After 14 mo.**	**After 18.6∼33.4 mo.**
	No calcification	No calcification	No calcification	No calcification
